# Risk based serological survey of Rift Valley fever in Tunisia (2017–2018)

**DOI:** 10.1016/j.heliyon.2021.e07932

**Published:** 2021-09-04

**Authors:** Sana Kalthoum, Elena Arsevska, Kaouther Guesmi, Aymen Mamlouk, Jamel Cherni, Monia lachtar, Raja Gharbi, Bassem Bel Haj Mohamed, Wiem Khalfaoui, Anissa Dhaouadi, Mohamed Naceur Baccar, Haikel Hajlaoui, Samia Mzoughi, Chédia Seghaier, Lilia Messadi, Malek Zrelli, Soufien Sghaier, Catherine Cêtre-Sossah, Pascal Hendrikx, Cécile Squarzoni-Diaw

**Affiliations:** a National Center of Zoosanitary Vigilance, Tunis, Tunisia; b CIRAD, UMR ASTRE, F-34395 Montpellier, France; c ASTRE, Univ Montpellier, CIRAD, INRAE, Montpellier, France; d Service de Microbiologie et Immunologie, Ecole Nationale de Médecine Vétérinaire de Sidi Thabet, Univ. Manouba, Sidi Thabet, Tunisia; e Ministère de l’Agriculture, Direction Générale des Services Vétérinaires, Tunis, Tunisia; f Institut de la Recherche Vétérinaire de Tunisie (IRVT), Tunis, Tunisia; g CIRAD, UMR ASTRE, F-97490 Sainte Clotilde, La Réunion, France

**Keywords:** QRA methodology, Risk mapping, Survey, Tunisia, Rift valley fever, Small ruminants, Camels

## Abstract

Rift Valley fever (RVF) has been reported in the sub-Saharan region of Africa, Egypt and Arabian Peninsula - Yemen and Saudi Arabia, over the past 20 years and is a threat to both the animal and human populations in Tunisia. Tunisia is considered as a high-risk country for the introduction of RVF due to the informal movements of diseased animals already reported in the neighboring countries. The objective of this study was to assess the status of RVF in small ruminants and camels in Tunisia. A risk-based serological survey was conducted to evaluate the presence of RVF based on spatial qualitative risk analysis (SQRA). Samples were collected from small ruminants (sheep and goats) (n = 1,114), and camels (n = 173) samples, belonging to 18 breeders in 14 governorates between November 2017 and January 2018. Samples were tested using an RVF specific multispecies competitive ELISA. Out of the 1,287 samples tested for the presence of RVF IgG antibodies by ELISA, only one positive sample 0.07% (1/1 287) was detected but not confirmed with the virus neutralization test (VNT) used for confirmation. So far, no RVF outbreaks have been reported in Tunisia and our study confirmed the absence of RVF in livestock up to January 2018. Further investigations are needed to confirm the RVF-free status of Tunisia today.

## 1 Introduction

Rift Valley fever (RVF) is a mosquito-borne zoonosis that affects humans and domestic ruminants (camelids, cattle, goats, and sheep) [1] caused by a virus of the *Phlebovirus* genus that belongs to the *Phenuiviridae* family. The virus was identified for the first time in 1930 in the Rift Valley in Kenya [2, 3]. Humans are infected by the RVF virus (RVFV) through contact with the blood or organs of infected animals during slaughter, or when handling infected animals, or through the ingestion of contaminated meat and raw milk [4]. Thus, staff working in slaughterhouses, laboratories and hospitals are the most exposed [5]. However, mosquitoes are the main vectors involved in the spread of RVFV during epidemics. The RVFV has been isolated from at least 40 mosquito species belonging to eight genera (mainly *Aedes* spp. and *Culex* spp.) [6, 7] when feeding on viremic animals. Infected females of *Aedes* spp. are known to transmit the virus to their progeny, via desiccated eggs that are resistant to drought, thus maintaining the viral life cycle [8].

The feeding activities of these mosquitoes rely mainly on environmental and climatic factors (rainfall, temperature) and outbreaks are likely to occur during heavy rainfall events in areas susceptible to flooding [9]. The mode of transmission varies with the ecosystem. For example, the most recent epidemics in Mayotte and Senegal showed that depending on the environmental context and on the typology of the farms, transmission of the vector or transmission linked to direct contact between herds and between animals can be of varying importance [10]. In infected livestock, the most common clinical signs are fever, massive abortions, high morbidity and mortality among young animals [11]. In humans, RVF causes a febrile and a hemorrhagic syndrome (epistaxis, hemoptysis, melena, hematemesis, gingival bleeding, bruising), and in severe cases, death [12].

The geographical distribution of RVF indicates that until 2000, the disease was limited to sub-Saharan Africa before expanding to the Arabian Peninsula [13]. As far as North African countries are concerned, Egypt experienced extensive outbreaks in 1977–78 and it is believed that the virus was introduced from Sudan through the Aswan dam [14]. Smaller epidemics occurred in 1993–94, 1996–97, followed by a larger outbreak in 2003. Serological surveys in animals and humans revealed the enzootic profile of the disease in Egypt [15]. In December 2019, Libya reported several RVF outbreaks in the southern part of the country [16]. As far as the North Africa are concerned, in 2008 and 2009, serological studies were conducted in Sahrawi refugee camps (Tindouf Province) on the south-western border with western Sahara (Algeria), in Mauritania, and in southern Morocco, in ruminants and human populations and RVF specific IgG antibodies were detected in camels and goats [17, 18].

In Tunisia, a serological survey was carried out in 2014 in the Centre of Tunisia (governorates of Sfax, Mahdia and Sousse) and revealed the presence of RVF specific IgG antibodies in human samples despite their absence in samples from febrile patients and slaughterhouse workers [19]. Additional RVF focused seroprevalence studies conducted on animal samples such as dromedaries in 2017 [20], goats and sheep in 2006–2007 [21] did not confirm active circulation of RVF in Tunisia. However, a study by Selmi et al. using targeted sampling reported 34% seroprevalence in camel populations in the southern governorates of Tunisia. This result could be explained by the fact that sampled camels may originate from illegal trade (Sudan, Chad and Niger), and may have been introduced into Tunisia through Libya [22].

Recent studies in Tunisia demonstrated that climatic factors might influence the distribution and abundance of the mosquitoes that transmit RVFV [23]. The mean temperature of the warmest quarter, the mean temperature of the coldest quarter, isothermally, and annual precipitation are considered to be the most significant climatic factors that influence vector distribution in risk areas [23]. The intensification of animal trade has also been shown to increase the risk of RVFV introduction and spread, and hence emergence in previously unaffected territories [24]. The latest epidemics have shown that, depending on environmental and livestock conditions, vector and direct transmission affect the magnitude of the epidemic differently [10]. A high-performance surveillance system is required for early detection of RVF outbreaks to avoid the economic losses that can result from emergence of the disease. The risk-based surveillance approach (RBS) can be used to improve detection of RVF as it is more sensitive, would provide higher positive predictive values, and enable more effective and efficient allocation of resources in countries with limited resources [25]. Indeed, this method considerably reduces the number of areas to be surveyed and hence the cost of targeted surveillance [25]. In Tunisia, a risk-based surveillance approach was implemented for foot and mouth disease (FMD) in 2017/2018 completed by a spatial risk analysis. A serological survey conducted in very-high and high-risk *imadas* helped estimate antibody prevalence to FMD [26]. In fact, Tunisia is at permanent risk of the introduction of several vector and non-vector borne infectious diseases, including RVF, due the illegal movement of small ruminants that is most intense during religious events [27].

The aim of this study was thus to investigate the circulation of RVF in the small ruminant and camel populations in Tunisia, using a serological cross-sectional survey in areas considered as high and very high-risk areas for the introduction of RVF.

## 2 Materials and methods

### 2.1 Period of study and risk-based survey

Tunisia is located in the northern eastern part of Africa between latitudes 30° and 38°N, and longitudes 7° and 12°E, covers 163,610 square km and had 11.7 million inhabitants in 2019 [28]. Administratively, it is organized in 24 governorates and 2,075 *imadas*. A risk-based survey targeting small ruminants and camels in the high and very-high risk areas was conducted in winter, i.e., between November 24, 2017 and January 30, 2018. The study area was represented by 23 randomly sampled *imadas* out of 841 *imadas* classified as high and very-high risk of exposure to RVF using a qualitative risk assessment (QRA) method [29]. The survey was limited to the two strata (very-high and high risk) for financial reasons.

The risk factors used to characterize the different levels of risk of exposure to RVFV included:

a) Ruminant density (number of animals per km^2^) [30],
b) Accessibility to other *imadas* (average travel time in minutes) [31],
c) Frequency of national and cross-border ruminant movements [32],
d) Presence/absence of permanent/temporary lakes and rivers, i.e., water bodies [33],
e) Abundance of five of the known competent mosquito vectors in Tunisia, i.e. *Culex pipiens*, *Aedes vexans*, *Aedes aegypti*, *Anopheles gambiae*, *Culex quinquefasciatus* [34].

**Figure 1:**
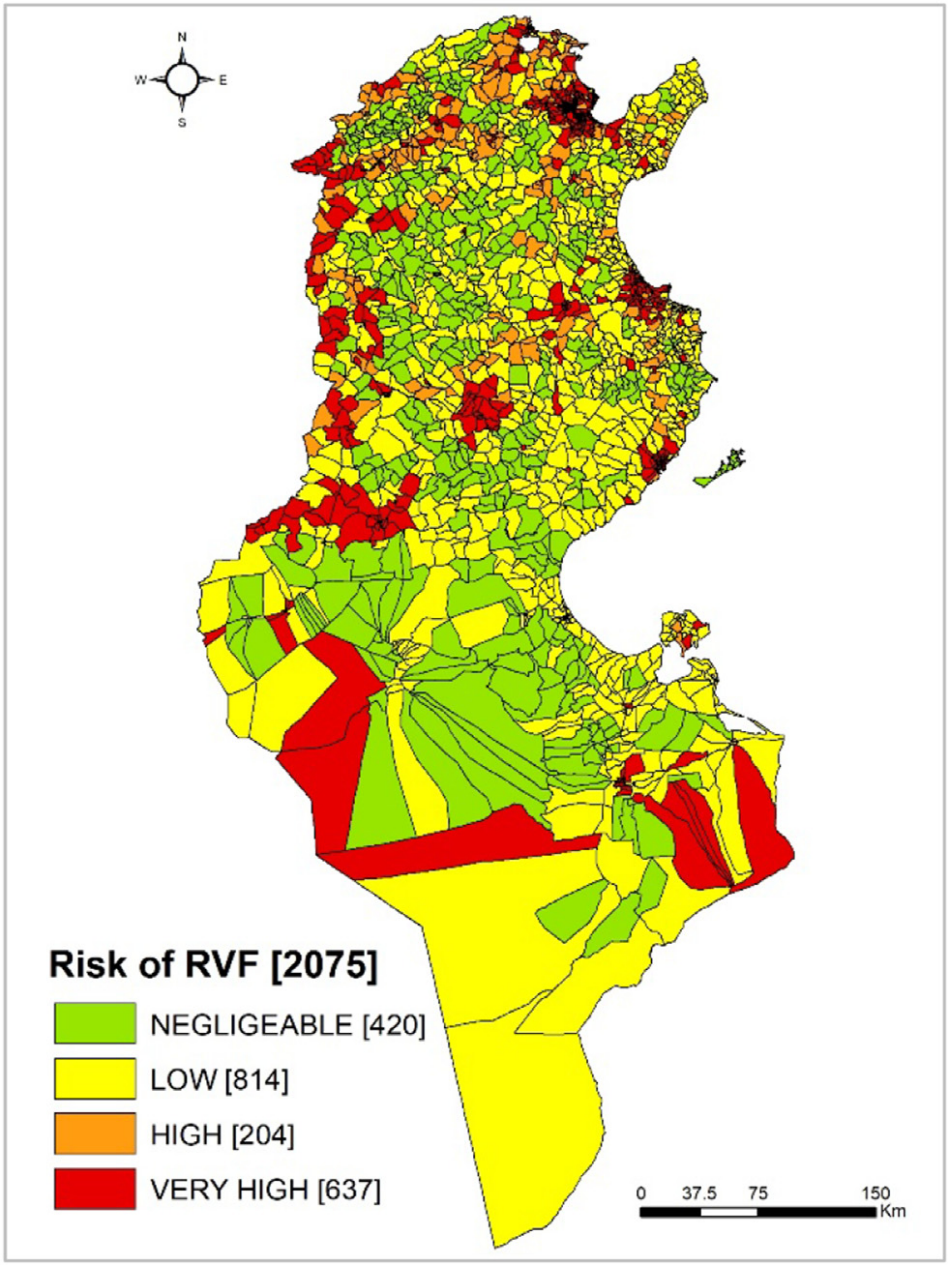
Risk map of RVF occurrence used to identify the high and very-high risk zones for risk-based sampling in small ruminants, Tunisia, 2017–2018.

Quantitative risk factors were categorized into quantiles and transformed into four classes (negligible, low, high, and very-high risk). Risk factors with presence/absence data were categorized in only two classes. Next, all risk factors were combined spatially using predefined Boolean combinations as described in the qualitative risk assessment method [29]. As a result, 204 and 637 *imadas* were qualified as respectively, high, and very-high risk areas out of a total of 2,075 *imadas* (Figure 1).

**Figure 2:**
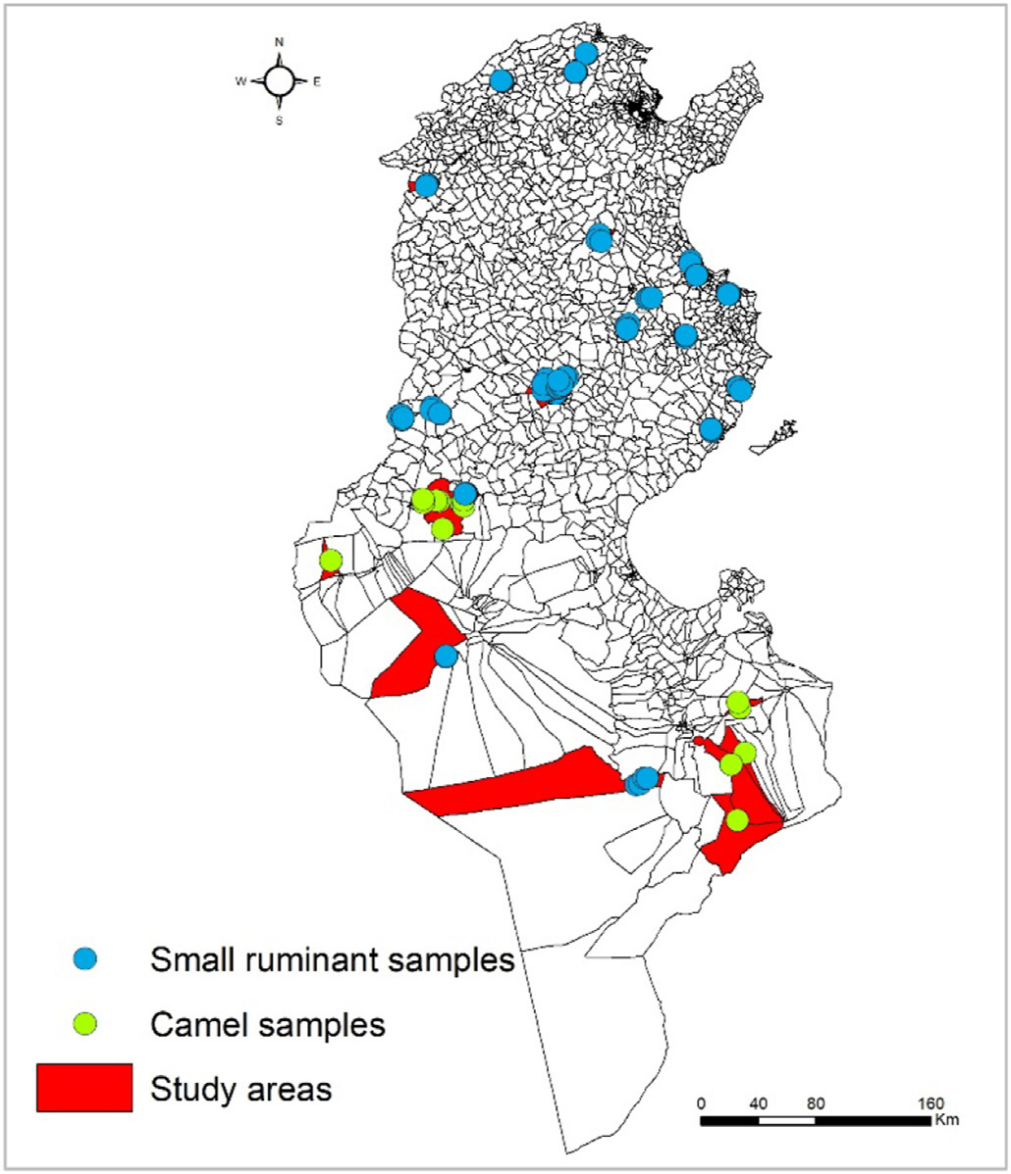
Study areas and geographical distribution of surveyed breeders of small ruminant and camels.

The number of *imadas* required to detect at least one RVF positive animal was calculated based on the absolute precision of 2.5%, risk error of 5% and expected prevalence (p) in the two risk strata (very high and high risk), thus giving:

- A very-high risk stratum with an expected prevalence rate p1 of 15% of infected *imadas.* There are 637 *imadas* in this stratum (31%).
- A high risk stratum with an expected prevalence rate p2 of 10% of infected *imadas.* There are 204 *imadas* in this stratum (10%),

Twenty-three *imadas* were randomly chosen from the 841 *imadas* found to be at high and very-high risk of exposure to RVF. Fifty animals belonging to five breeders per *imada* were randomly selected. A total of 1,150 small ruminant sera were sampled from the 23 very-high and high risk selected *imadas* located in 13 governorates (Figure 2).

### 2.2 Sampling of small ruminants and camels

The sampling of small ruminant populations involved a three-stage purposive selection of *imadas*, breeders and animals. *Imadas* were randomly sampled using Excel. In the 23 sampled *imadas*, the snowball sampling method was used to select the small ruminant breeders [35]. The concept of this method is as follows: from the first breeder, the investigator accesses the following one, thus proceeding to successive contacts. The first selected breeder answered the questions and then suggested other breeders to be surveyed according to the investigator's criteria. Random sampling was used to select animals at the breeder's premises. Ten animals per breeder were included in the study.

One-humped camels (*Camelus dromedarius*) are distributed in the southern part of Tunisia, which consists of six governorates and 382 *imadas*, where high densities of camels have been recorded (Figure 2). The total camel population in the six governorates is estimated at 40,868 camels [36]. Three-stage purposive selection was used to select the *imadas*, breeders and camels. The required numbers of *imadas* to detect at least one camel positive for RVF was calculated based on the absolute precision of 2.5%, risk error of 5% and an expected prevalence (p) of 2% since no evidence of RVFV circulation in this species was reported in previous studies in Tunisia. A total of 228 samples of camel sera were thus required.

Five and 10 ml of whole blood was collected in Vacutainer tubes (Becton Dickinson, USA) from the jugular vein of small ruminants and camels, respectively. Samples were allowed to clot at 15 °C and serum was separated from whole blood by centrifugation; sera were stored at -20 °C in the laboratory.

### 2.3 Data collection and analysis

Data on breeders and sampled animals were collected in a face-to-face interview during the risk period for RVF (November 2017–January 2018). This period corresponds to the period when the vector is most abundant when the animals are gathered in pastures, corresponding to increased risk. A pretested semi-structured questionnaire was used to collect the data. It included two parts:1) information on the farm (governorate, *imada*, GPS coordinates, date of the survey, owner's name, address, number of employees, animal species present and number of animals of each species), 2) information on the sampled animal (species, identification, age, sex, breed, and abortion history).

The collected data were entered into the Access database and descriptive statistics were performed using R open-source programming language (version 3.5.2) and Epinfo software version 3.5. P-values lower than 0.05 were considered statistically significant at confidence level of 95%. Maps were created using ArcGIS software version 10.3.

### 2.4 Detection of RVFV specific antibodies

Sera were tested using the ID Screen® Rift Valley Fever competition multi-species ELISA kit (ID.vet, Grabels France) according to the manufacturer's instructions. This competition test detects antibodies of all types of animal species and its diagnostic specificity is estimated at 100% while diagnostic sensitivity ranges from 91-100% [37]. The optical density (OD) of samples was measured at 450 nm wavelength using a spectrophotometer microplate reader (Multiskan™ FC, Thermo Fisher Scientific™, USA) and the results were calculated according to the following formula: S/N (%) = (OD sample/OD negative control) x 100, where S is the tested sample, and N is the negative control. Serum samples with S/N values lower than 40% were considered positive, doubtful if the values were between 40% and 50 %, and negative if they were higher than 50%. Samples that tested positive with cELISA were specifically analyzed with the virus neutralization test (VNT), the test recommended by the OIE [38] to detect and confirm the presence of RVFV neutralizing antibodies.

## 3 Results

### 3.1 Characterization of the surveyed farms and animals

According to the sampling protocol, 1,150 small ruminant samples and 228 camel samples were necessary to detect the expected RVF prevalence. Because of field constraints, it was only possible to collect 1,114 small ruminant samples (from 112 breeders) and 173 camel samples (from 18 breeders) during the study period (Supplementary material 1).

Of the 112 breeders of small ruminants included in the survey, 98.2% (110/112) were private farms and only two were public farms (Table 1). Of the camel breeders, 61.1% (11/18) were private and 38.9% (7/18) were transhumant farms. The collected data showed that abortions were reported by 65.1% (73/112) of the farms surveyed in the past year compared to 34.9% (39/112) of farms with no history of abortions (Table 2). Respectively, 91.3% (1,017/1,114) and 100% (173 out of 173) of the sampled animals in the small ruminant and camel categories were females. The age of the sampled small ruminants ranged between 2 months and 15 years with a median of 3 years. The minimum-recorded age of the sampled camels was 1 year and the maximum was 15 years, with a median of 6 years. As shown in Table 3, the age group 2–4 years was the most common, 54% (607/1,114), among the small ruminants. Among the camels, the most common age group was 9–15 years (37.4%).

**Table 1:** Categorization of the surveyed farms (public and private).

Type of farms	Small ruminants	Camels
Public	2 (1.8%)	0 (0%)
Private	110 (98.2%)	11 (61.1%)
Transhumant	0 (0%)	7 (38.9%)
Total	112	18

**Table 2:** Occurrence of abortion on the surveyed farms.

Occurrence of abortion in the past year	Number of small ruminant farms	Number of camels farms
No	39 (34.9%)	8 (44.4%)
Yes	73 (65.1%)	10 (55.6%)
Total	112	100

**Table 3:** Age classification of the animals in the serological survey of RVF.

Age class	Small ruminants	Camels
≤2 years	334 (29.9%)	51 (29.8%)
(2–4]	607 (54.5%)	13 (7.6%)
(4–9]	157 (14.09%)	43 (25.1%)
(9–15]	16 (1.4%)	64 (37.4%)
Total	1114 (100%)	171 (100%)

### 3.2 Detection of RVFV specific antibodies

**Figure 3:**
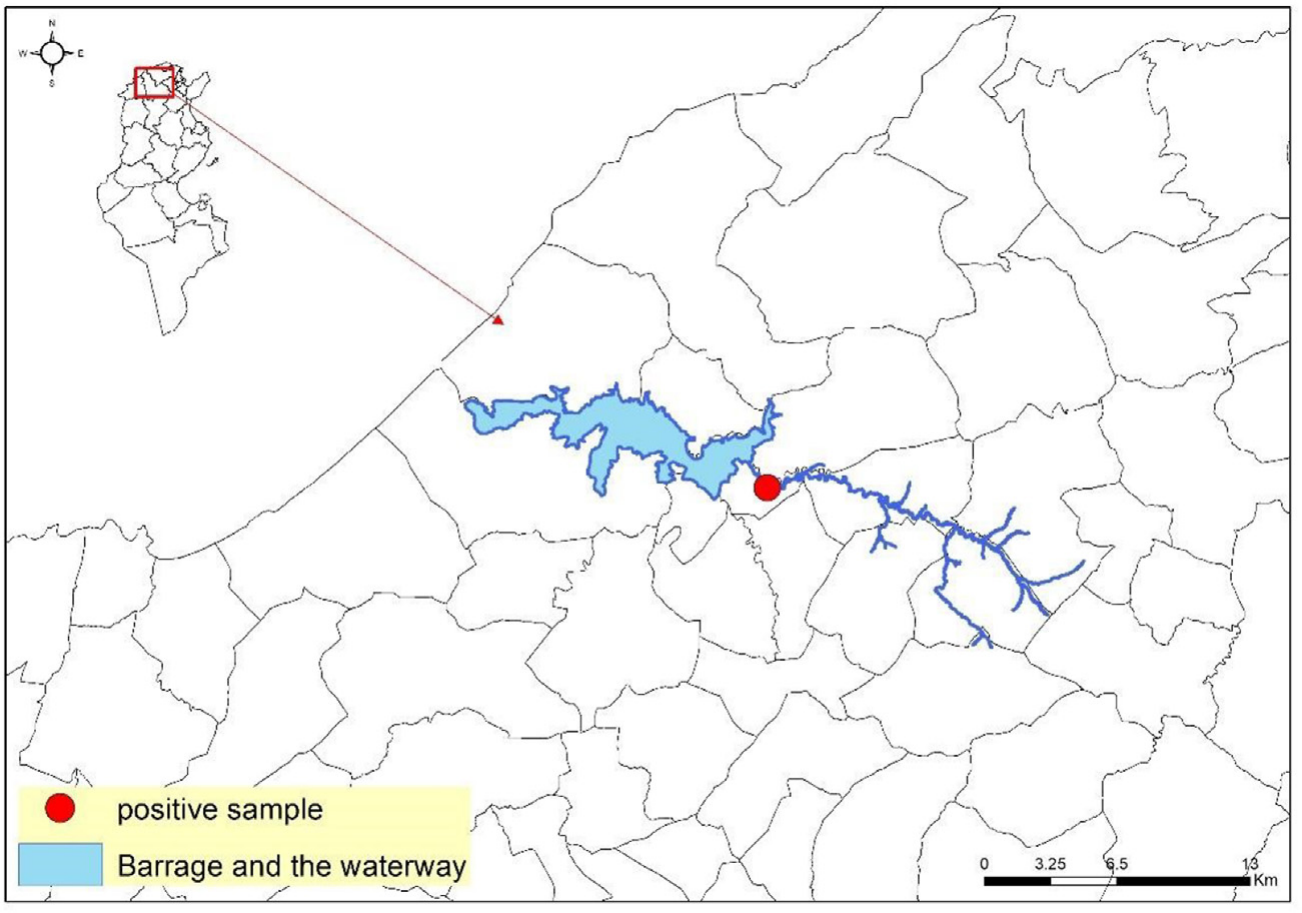
Location of the cELISA RVF seropositive detected sample, showing its proximity to a dam and a waterway.

Of the 1,287 samples tested for the presence of RVF antibodies, only one positive sample (0.07%) was detected in a 3-year-old ewe from the *imada* of Nefza East (governorate of Beja located in northeastern Tunisia). The sheep belonged to a farm located at a distance of 1.5 km from a dam and 0.12 km from a natural waterway with abortion reported on the farm (Figure 3). The sample tested positive with ELISA but negative with the virus neutralization confirmatory test (VNT).

## 4 Discussion

The aim of this study was to investigate the circulation of RVFV in small ruminant and camel populations in Tunisia using a risk-based sampling method, supported by a spatial qualitative risk analysis. RVF is known to be a vector-borne and zoonotic disease and its emergence depends on climatic factors, mainly rainfall, and is mainly transmitted by *Aedes* and *Culex* [39, 40]. Recent changes in the epidemiological situation of RVF have often been linked to the increasing density in sheep and cattle, animal mobility, intensification of the livestock trade and climatic factors [41]. Previous studies in Tunisia revealed ubiquitous distribution of *Culex* and *Aedes* species [42]. Other studies demonstrated the RVF competence of *Culex pipiens* tested using ZH548 and Clone 13 viral strains under laboratory conditions and reported a transmission infection rate of 14.7%. This species would likely involved in the spread of RVFV if the virus were introduced in Tunisia [43]. The poorly controlled cross-border movement with neighboring countries (Algeria, Libya) in the presence of the potential vectors puts Tunisia at permanent and high risk of the introduction and spread of RVF [43].

Several studies that assess the risk of introduction and the spread of RVF in Tunisia, revealed that the northern and central-eastern regions are likely to be the most suitable regions for RVF incursion and epizootic occurrence [44] but no risk-based serological survey was undertaken based on the results of the risk assessment to confirm this hypothesis.

Our study is the first investigation of RVFV circulation in Tunisia using RBS methodology. Samples were selected based on the risk of exposure to RVF including the risk of introduction and risk of spread of RVF (animal density, vector abundance, accessibility of *imadas* and movements of small ruminants at national level) [29]. The RBS approach was used in this study improve surveillance of RVF in Tunisia. To confirm freedom from the disease, veterinary services could reduce the normally required sample size using risk-based samples, especially when financial resources are limited [45, 46].

One limitation of our study was that samples came from farms where the animals were not identified, meaning tracing was not possible in the case of positive results.

The RVF serological investigations conducted in this study revealed the presence of only one seropositive sheep despite the use of a highly sensitive and specific ELISA. This sample was negative using the viral neutralization test (VNT), thereby confirming RVF was not circulating in Tunisia in 2017–2018 among the animals sampled. The sheep farm where the cELISA positive sample was detected is located in Nefza (governorate of Beja) and abortions were already reported in this farm before the survey. Further epidemiological investigations in this *imada* are thus needed to confirm its RVF free status.

Regarding camel sampling, the required number of 228 samples was not reached due to the free-living livestock system where the herds remain for a long period without herders and are waited for at fixed water points, often in the birthing season or in the period of health checks [47]. In a previous study conducted in Tunisia by Fakhfakh et al. (2006 and 2007), 610 samples randomly collected from animals near water sources were all found to be RVF seronegative [21]. In 2017, Ben Hassine et al. did not find any RVF seropositive samples in camels (n = 118) in the southern region of Tunisia [20]. However, a serological survey carried out in the summer of 2014 in the governorates of Sousse, Sfax and Mahdia (east-central Tunisia) indicated that among the 181 sera of human patients suffering from a febrile episode, 14 were RVF IgM positive. This result pointed to recent circulation of the RVF virus in Tunisia. The authors reported they were unable to establish a link between these employees and the slaughter of animals directly imported from abroad [19]. Recently, Selmi et al. demonstrated that 162 camels out of 470 were found to be antibody positive to RVF using the same competitive ELISA and suggested that other tests should be carried out, such as the virus neutralization test to confirm the presence of this disease in Tunisia [22]. The presence of positive camels could be explained by the fact that animals with RVFV antibodies may originate from different sources (illegal trade, different livestock markets) [22]. Based on this result, the circulation of RVFV in Tunisia requires additional confirmatory tests such as the viral neutralization test (VNT), to confirm the presence or the absence of the disease in ruminants in some of the governorates in Tunisia bordering other countries involving possible illegal animal movements.

In the present study, most of the small ruminants sampled were sedentary and thus not at risk of exposure to the introduction of the disease through commercial movements and are not mixed with other herds, which would increase the probability of infection. However, this result should be interpreted with caution since RVF was detected in Algeria in 2008 and Morocco in 2009 [48], and zones that are suitable for the RVF vector were identified [49]. In this context, we recommend further serological investigations of farms that report abortions and high offspring mortality in camels and small ruminants, especially in the governorates that border Libya and Algeria. We also suggest implementing the RBS in addition to conventional surveillance (event and active surveillance) in Tunisia. This type of surveillance should target the border sectors and zones with uncontrolled animal movement. The RBS needs to be conducted in farms raising non-sedentary small ruminants (with abortions and high offspring mortality) or in camels in the bordering governorates, during the high risk periods of the RVF occurrence due to vector abundance (in autumn when there is a significant increase in rainfall and during the Eid el-Kebir religious festival) [50, 51]. We are of the opinion that implementing the RBS test in Tunisia in addition to the conventional (event and active) surveillance would increase the vigilance and improve early detection of outbreaks, especially since the country has limited resources (human resources, logistical resources, diagnostic kits, etc.). To optimize surveillance and control of the disease in high and very-high risk areas, veterinary services could implement control measures such as vaccination of animals in livestock markets and check illegal movements of animals. Surveillance of RVF should also focus on data collected on suspicions or confirmed cases in humans in order to implement the same surveillance strategies and actions in the field. As recommended by the world organization for animal health (OIE), RVF detection capacity can be further increased through the one health approach by implementing multidisciplinary collaboration and integrated surveillance involving both public and animal health.

## 5 Conclusion

The risk-based methodology, supported by risk mapping, is a very useful tool in veterinary medicine that would help authorities understand the epidemiology and the risk of the disease occurrence better. This methodology provides more details on risk areas, information that is essential for the design of disease prevention and surveillance. The risk-based methodology identifies target regions where more specific activities of surveillance need to be implemented. In Tunisia, risk-based mapping is increasingly used in veterinary medicine because the risk of introduction and risk of spread of diseases is very high due to the geographical position of the country and the permanent illegal animal movements between Tunisia and neighboring countries.

## Declarations

### Author contribution statement

Sana Kalthoum: Conceived and designed the experiments; Analyzed and interpreted the data.

Elena Arsevska, Chédia Seghaier, Pascal Hendrikx, Cécile Squarzoni-Diaw: Conceived and designed the experiments; Wrote the paper.

Kaouther Guesmi, Jamel Cherni, Monia lachtar, Raja Gharbi, Bassem Bel Haj Mohamed, Wiem Khalfaoui, Anissa Dhaouadi, Mohamed Naceur Baccar, Haikel Hajlaoui, Samia Mzoughi: Performed the experiments.

Aymen Mamlouk, Soufien Sghaier, Catherine Cêtre-Sossah, Conceived and designed the experiments; Analyzed and interpreted the data; Contributed reagents, materials, analysis tools or data.

Lilia Messadi: Contributed reagents, materials, analysis tools or data; Wrote the paper.

Malek Zrelli: Analyzed and interpreted the data; Wrote the paper.

### Funding statement

This research did not receive any specific grant from funding agencies in the public, commercial, or not-for-profit sectors.

### Data availability statement

Data will be made available on request.

### Declaration of interests statement

The authors declare no conflict of interest.

### Additional information

No additional information is available for this paper.

## Appendix A Supplementary data

Farm coordinates Farm coordinates
